# In Silico Modeling of Liver Metabolism in a Human Disease Reveals a Key Enzyme for Histidine and Histamine Homeostasis

**DOI:** 10.1016/j.celrep.2016.05.014

**Published:** 2016-05-26

**Authors:** Roberto Pagliarini, Raffaele Castello, Francesco Napolitano, Roberta Borzone, Patrizia Annunziata, Giorgia Mandrile, Mario De Marchi, Nicola Brunetti-Pierri, Diego di Bernardo

**Affiliations:** 1 Telethon Institute of Genetics and Medicine, 80078 Pozzuoli, Italy; 2 Medical Genetics, San Luigi University Hospital, 10043 Orbassano, Italy; 3 Department of Clinical & Biological Sciences, University of Turin, 10043 Orbassano, Italy; 4 Department of Translational Medicine, Federico II University, 80131 Naples, Italy; 5 Department of Chemical, Materials and Industrial Engineering, Federico II University, 80125 Naples, Italy

## Abstract

Primary hyperoxaluria type I (PH1) is an autosomal-recessive inborn error of liver metabolism caused by alanine:glyoxylate aminotransferase (AGT) deficiency. In silico modeling of liver metabolism in PH1 recapitulated accumulation of known biomarkers as well as alteration of histidine and histamine levels, which we confirmed in vitro, in vivo, and in PH1 patients. AGT-deficient mice showed decreased vascular permeability, a readout of in vivo histamine activity. Histamine reduction is most likely caused by increased catabolism of the histamine precursor histidine, triggered by rerouting of alanine flux from AGT to the glutamic-pyruvate transaminase (GPT, also known as the alanine-transaminase ALT). Alanine administration reduces histamine levels in wild-type mice, while overexpression of GPT in PH1 mice increases plasma histidine, normalizes histamine levels, restores vascular permeability, and decreases urinary oxalate levels. Our work demonstrates that genome-scale metabolic models are clinically relevant and can link genotype to phenotype in metabolic disorders.

## Highlights

• In silico model of liver metabolism reveals global metabolic alterations in PH1
• Changes in amino acid metabolism in PH1 result in a reduction of histidine and histamine
• GPT overexpression normalizes histamine levels and reduces oxalate in PH1 mice

## Graphical Abstract

**Figure:**
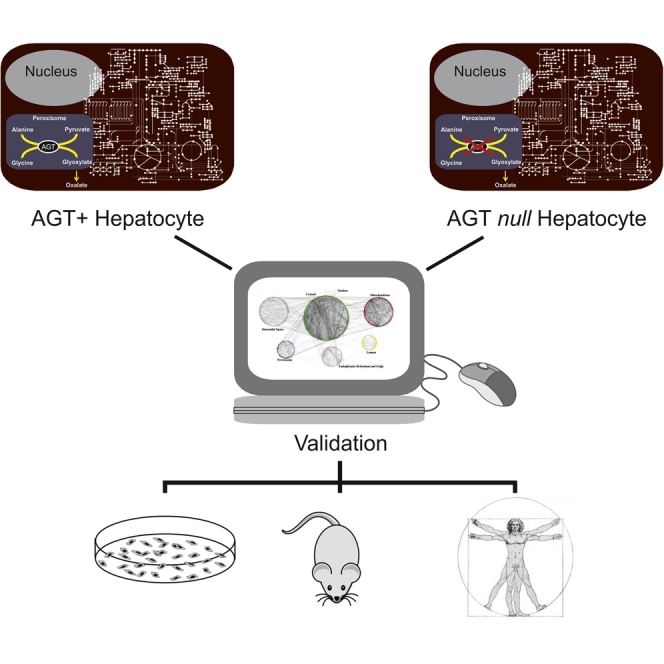

## Teaser

Pagliarini et al. use a computational model of liver metabolism to predict alterations caused by the loss of alanine:glyoxylate aminotransferase (AGT), resulting in primary hyperoxaluria type I (PH1). In addition to known disease biomarkers, the model predicts a reduction in histidine and histamine levels. GPT overexpression in PH1 mice normalizes histamine and oxalate levels.

## Introduction

Metabolism is primarily or secondarily affected in several acquired and inherited human diseases. Characterization of the metabolic changes occurring in health and disease states has a wide range of implications, from elucidation of pathogenetic mechanisms to development of new biomarkers and drug discovery.

Inborn errors of metabolism (IEMs) are a group of Mendelian disorders resulting from genetic disruption of single metabolic enzymes. A large number of these reactions occurs in the liver. The study of these disorders has been instrumental to understanding the physiological consequences of metabolic reactions and pathogenesis of more common multifactorial diseases. In contrast to Mendelian diseases, which are due to severe impairment of single-enzyme reactions, common multifactorial diseases may result from mild impairment of several metabolic reactions (Lanpher et al., 2006). Nevertheless, our understanding of the consequences of single-enzyme deficiencies on metabolism as a whole are underappreciated, since most studies have been narrowly focused on the affected metabolic reactions, thus neglecting alterations of more distant metabolites. In most patients affected with IEMs, there are few therapeutic options that are often limited to common sense interventions aimed at either reducing the substrate or increasing the product of the affected reaction.

Tissue-specific genome-scale metabolic models, which have only recently become available through the efforts of the modeling community, allow in silico prediction of the effects of genetic or chemical perturbations on human metabolism (Gille et al., 2010, Jerby et al., 2010, Shlomi et al., 2009, Thiele et al., 2013). These computational models have been used to predict, for example, cancer drug targets (Folger et al., 2011), anti-aging drugs (Yizhak et al., 2014), and biomarkers for rare metabolic disorders (Duarte et al., 2007, Shlomi et al., 2009, Thiele et al., 2013).

Here we applied a computational approach to predict and analyze the metabolic alterations occurring in hepatocytes lacking alanine:glyoxylate aminotransferase (AGT), a peroxisomal enzyme encoded by the *AGXT* gene and mutated in primary hyperoxaluria type 1 (PH1).

PH1 is an autosomal recessive disease that presents with hyperoxaluria, progressive renal involvement, and systemic deposition of calcium oxalate in multiple organs and tissues. Although the enzyme is only expressed in hepatocytes, lack of AGT results in excessive production of oxalate by the liver, leading to oxalate-induced damage in several tissues, particularly in kidneys. PH1 is a severe disease that results in high morbidity, pain, disability, poor quality of life, and early death if treated late or left untreated. Effective treatments for PH1 are still lacking and combined liver-kidney transplantation is the only available therapeutic option for patients with severe forms (Hoppe et al., 2009).

PH1 was chosen for our computational metabolic modeling for the following reasons: (1) the defective enzyme is expressed only in the liver, (2) enzyme deficiency does not result in structural changes of the liver that could secondarily affect metabolic functions, and (3) a mouse model is available to readily investigate alterations predicted by the computational approach (Salido et al., 2006). We demonstrated in vitro, in vivo, and in the sera of PH1 patients that current genome-scale metabolic models are now sufficiently detailed to enable in silico prediction of the pathophysiologic consequences of a single-enzyme deficiency and to suggest therapeutic targets. We correctly identified metabolites known to accumulate in PH1, such as glyoxylate, glycolate, and oxalate, but also discovered a link between AGT and the metabolism of histidine and histamine. Moreover, we proved that such models can be used to identify and validate potential therapeutic enzyme targets for PH1.

## Results

### Computational Approach to Predict Metabolic Alterations Due to Single-Enzyme Defect in Hepatocytes

We applied a computational systems-level approach to simulate in silico metabolic alterations in hepatocytes due to the gain of function (GoF) or loss of function (LoF) of a single enzyme starting from a recently published genome-scale metabolic network model of human hepatocytes (HepatoNet1). The in silico model comprises 777 metabolites and 2,539 reactions across eight compartments (Gille et al., 2010).

We first extended HepatoNet1 by including 50 additional enzymatic and transport reactions and eight metabolites (Table S1) related to glyoxylate metabolism that were needed to model PH1, including the reaction carried out by AGT. One of the reactions we added is the *cytosolic* alanine-glyoxylate transamination carried out by the glutamic-pyruvate transaminase (GPT), also known as alanine aminotransferase (ALT) (Donini et al., 2009). Hence, in the extended model, GPT catalyzes in the cytoplasm the same reaction as AGT in the peroxisome, in addition to its canonical function (glutamate-pyruvate or alanine-transaminase), already present in the original model.

We next applied an algorithm we recently proposed, differential flux-balance analysis (DFA), to predict differences in metabolic fluxes and metabolite levels between wild-type (WT) and single-enzyme-defective hepatocytes across 442 different metabolic objectives (Table S2) (Pagliarini and di Bernardo, 2013). Of these, 123 simulate physiological hepatic functions (e.g., ATP production and amino acid degradation), while the remaining 319 are used for model validation (Gille et al., 2010).

DFA is based on flux balance analysis (FBA), a standard mathematical procedure to semiquantitatively estimate the metabolic flux of each metabolic reaction in a genome-scale model at steady state (i.e., no accumulation or depletion of metabolites) when satisfying a given metabolic objective (e.g., consume amino acids to produce glucose) (Orth et al., 2010). For each metabolic objective, DFA computes the difference between the metabolic fluxes predicted by FBA in the WT genome-scale model versus the perturbed model (LoF or GoF of a single enzyme or of a set of them). The LoF model lacks the enzyme under investigation, whereas the GoF model is constrained so that the reaction(s) carried out by the enzyme of interest is forced to be more active than in the WT. The end result is a ranked list of metabolic fluxes of all the reactions in the model (2,589), sorted by their difference in the LoF (or GoF) model versus the WT model. This difference is computed as the average across the 442 metabolic objectives. Hence, the reactions that are most affected by the LoF (or GoF) will be found at the top of the ranked list. Once these differential metabolic fluxes have been identified, the metabolites (785) involved in the associated reactions are ranked to predict the most affected ones. This is done, for each metabolite, by summing the contribution of all the differential fluxes involving the same metabolite. This value is then used for the ranking, so that the metabolites whose fluxes are most affected by the LoF (or GoF) will be found at the top of the ranked list. Finally, we applied a modified version (Experimental Procedures) of flux variability analysis (FVA) (Duarte et al., 2007, Shlomi et al., 2009, Thiele et al., 2013) to the top-ranked metabolites to estimate whether these tend to increase or decrease in the LoF (or GoF) model compared to the WT model.

### In Silico Model of PH1

We applied DFA and FVA on the extended HepatoNet1 model to simulate the effect of AGT enzyme LoF, causative of PH1. We thus obtained a ranked list of 590 metabolites (Table 1; Table S3), obtained from the list of 785 metabolites after removing small molecules, co-factors, and pooled metabolites in Table S2, and a list of 2,589 reactions (Table S3) ranked according to their predicted change following AGT LoF. We observed that hundreds of reactions, which are not directly related to peroxisomal AGT, are perturbed throughout the metabolic network and across multiple cellular organelles (Table S3). Interestingly, the AGT-like glyoxylate detoxification reactions carried out by AGXT2 in mitochondria and GPT in the cytoplasm increased their flux following AGXT LoF (Table S3; Figure S1). However, these compensatory effects were not sufficient to normalize oxalate levels (Table 1; Table S3), in agreement with disease development in affected patients.

The significance of model predications was assessed by metabolite set enrichment analysis (MSEA) (Xia and Wishart, 2010). MSEA is a statistical procedure for metabolomic studies that takes as input a list of altered metabolites in a patient and automatically predicts the most likely metabolic disorder. This is done by checking whether the metabolites in input are statistically enriched for known biomarkers of the disease. The top 50 metabolites predicted to change the most by the in silico model of PH1 (Table S3) were used to run MSEA. MSEA ranked PH1 as the most likely disease of 346 diseases (Table S3, p value = 0.00117).

The metabolites predicted to change the most by the in silico analysis (i.e., ranked in the top 50 positions, Table S3) included all the three major urine and plasma biomarkers of PH1 patients (Table 1; Table S3), i.e., the disease-causing oxalate, glycolate, and glyoxylate (Danpure, 2006). In addition to the known disease biomarkers, several other metabolites listed in Table 1 were expected to change: pyruvate, alanine, and glycine are directly involved in the AGT reaction; hydroxypyruvate is oxidized to glycerate by glyoxalate reductase/hydroxypyruvate reductase (GRHPR), the enzyme deficient in primary hyperoxaluria type 2 (PH2) that catalyzes the reduction of glyoxylate to glycolate (Cramer et al., 1999); serine also was expected to change because of the serine-pyruvate transaminase activity of AGT (Danpure, 2006).

In contrast, other metabolites such as glutamate and α-ketoglutarate (aKG) were unexpected, but the most surprising and interesting metabolites were histamine and its precursor histidine, whose levels were predicted to be altered in the AGT LoF model. Histamine is a biogenic amine with central roles in allergic responses and gastric acid secretion, involved in regulation of immune response, inflammation-associated carcinogenesis, and neurotransmission. Histamine is endogenously generated from its precursor histidine by the histidine-decarboxylase in several tissues (Rosenthaler et al., 1965), including liver (Tran and Snyder, 1981), and this reaction was already present in HepatoNet1. So far, no relationship between histidine, or histamine, and AGT deficiency in PH1 has been reported.

### Histidine and Histamine Are Reduced in PH1

We measured liver and serum levels of aKG, histamine, and histidine in the *Agxt*^−/−^ mouse model of PH1 that recapitulates the biochemical features of PH1 patients (Salido et al., 2006) to validate the in silico model, which predicted non-obvious changes in these three metabolites (Table 1). Consistent with the in silico result (Table 1), aKG was significantly reduced in *Agxt*^−/−^ mouse serum (Figure S2A) compared to control mice.

**Figure 1:**
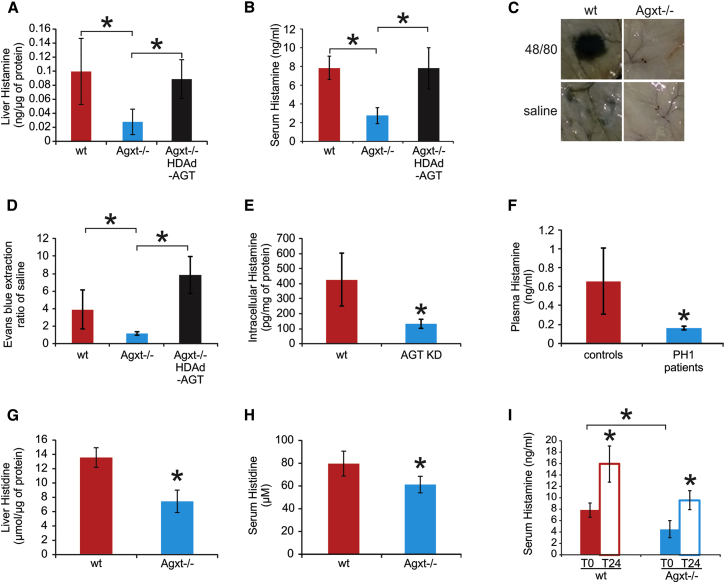
Reduction of Histamine in PH1 Mice and Patients and in Cells Knocked Down for AGT (A and B) Histamine levels in livers (A) and sera (B) were reduced in Agxt^−/−^ mice compared to wild-type (WT) mice (at least n = 3 per group). Agxt^−/−^ mice with vector-mediated expression of AGT protein showed increased histamine in both livers (A) and serum (B). (C) Compared to WT mice, Agxt^−/−^ showed reduced Evans blue extravasation in turned-over skin after intravenous injection of Evans blue followed by intradermal injections of either saline or compound 48/80. (D) The ratio of fluorescence units given by intradermal injection of 48/80 and normal saline was reduced in Agxt^−/−^ mice (n = 8 per group). (E) AGT-knockdown Huh-7 cells (AGT KD, Figure S3B) had reduced intracellular histamine compared to WT cells. (F) Plasma histamine levels were reduced in PH1 patients harboring *AGXT* mutations (Table S2; n = 3) compared to normal subjects (n = 5). (G and H) Histidine levels were reduced in livers (G) and sera (H) of Agxt^−/−^ mice compared to WT. (I) Intraperitoneal injections of histidine resulted in increased histamine levels at 24 hr after the injections (T24) compared to baseline levels (T0) in both Agxt^−/−^ and control WT mice (n = 6 per group; ^∗^p < 0.05). See also Figures S1, S2, and S3.

Histamine levels in liver and serum were also significantly reduced in *Agxt*^−/−^ mice compared to WT mice (Figures 1A and 1B). Histamine levels were increased compared to control uninjected *Agxt*^−/−^ mice (Figures 1A and 1B) when hepatic AGT expression was restored in *Agxt*^−/−^ mice by intravenous injection of a helper-dependent adenoviral vector encoding AGT under the control of a liver-specific promoter (HDAd-AGT) (Brunetti-Pierri and Ng, 2011, Brunetti-Pierri et al., 2005, Castello et al., 2016) (Figure S3A). Histamine levels were unaffected in *Agxt*^−/−^ mice injected with HDAd-AFP, a vector expressing the unrelated reporter gene α-fetoprotein under the control of the same expression cassette (serum histamine: 2.14 ± 0.85 ng/ml; n = 5). In addition, compared to WT controls, *Agxt*^−/−^ mice had decreased vascular permeability, a functional readout of histamine-mediated response, which was restored by liver-directed *AGXT* gene transfer (Figures 1C and 1D). Decreased histamine levels were also detected in *AGXT* knockdown human Huh-7 hepatic cells (Figure 1E; Figure S3B; Experimental Procedures) and in plasma of PH1 patients with confirmed *AGXT* mutations (Figure 1F; Table S3).

Histidine, the histamine precursor, was also significantly reduced in *Agxt*^−/−^ mouse liver (Figure 1G) and serum (Figure 1H) compared to control mice, consistent with the in silico results (Table 1). However, histamine concentration in normal human plasma is in the nanomolar range, whereas histidine concentration is in the micromolar range. Therefore, it is not obvious that a reduction in histidine levels should result in systemic reduction of histamine levels. To address this issue, we performed intraperitoneal injections of histidine in both WT and *Agxt*^−/−^ mice that resulted in increased serum histamine levels when compared to baseline levels (Figure 1I). This result confirms that changes in systemic histidine concentration can affect serum histamine levels in mice despite their great difference in plasma concentrations, as reported also in previous studies (Yoshikawa et al., 2014).

### AGT LoF Affects the Histidine Degradation Pathway

The in silico PH1 model revealed unexpected changes in histidine and histamine levels as a consequence of AGT LoF, which we confirmed both in vitro and in vivo.

**Figure 2:**
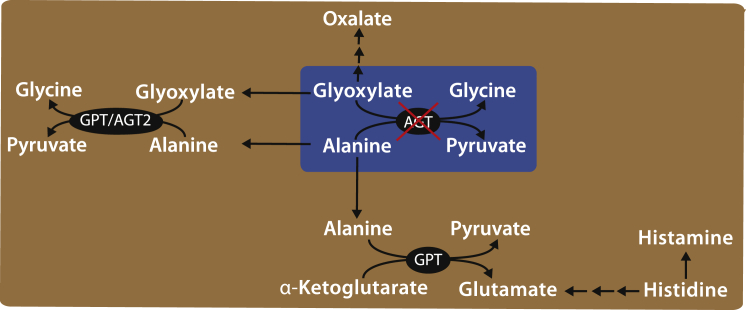
The In Silico Metabolic Model Reveals a Link between AGT LoF and Histidine Metabolism Mediated by the Action of GPT Both AGT- and GPT-mediated reactions involve alanine, glyoxylate, glycine, and pyruvate. GPT catalyzes two different reactions in heptocytes: the non-canonical glyoxylate detoxification reaction and the canonical reversible transamination between alanine and α-ketoglutarate to generate pyruvate and glutamate. Glutamate is a key compound in amino acid degradation. Alteration of metabolic fluxes caused by AGT LoF are thus propagated via GPT to histidine metabolism. Histidine can be metabolized to glutamate and α-ketoglutarate via a step of intermediary reactions (double arrows) and/or to histamine. The conversion of histidine into histamine also occurs in other cell types. See also Figure S1 and Tables S1, S2, S3, and S5.

The in silico analysis revealed a global perturbation of metabolism throughout the network, thus several different reactions might contribute to this effect. To better elucidate the most relevant pathways, we again applied MSEA to the metabolites most affected by AGT LoF in Table 1, but this time to investigate whether they were significantly enriched in known metabolic pathway(s) (rather than disease biomarkers) (Experimental Procedures). As shown in Table S3, the most significant pathways (p < 0.05) included amino acid metabolism (alanine, glycine, glutamate, and histidine), glutathione metabolism, ammonia recycling, and urea cycle. These last two pathways are involved in histidine degradation and glutamate metabolism. In addition, we noticed that aKG, alanine, pyruvate, and glutamate, all present in Table 1, are the metabolites involved in the alanine-transamination reaction (aKG + alanine → pyruvate + glutamate) carried out by GPT, the most abundant enzyme in liver, with a central role in glutamate metabolism (Figure 2). Moreover, according to the in silico model, the GPT-mediated alanine-transamination reaction increases its flux following AGT LoF (Figure S1; Table S3; Supplemental Experimental Procedures).

We next asked why AGT LoF should affect the flux through GPT and in turn affect histidine metabolism. As shown in Figure 2 and Figure S1 and detailed in the Supplemental Experimental Procedures, according to the model, the fluxes through GPT increase because alanine is no longer consumed by AGT in the peroxisome and it is thus available to GPT in the cytoplasm (Figure 2; Figure S1; Table S3). Hence, both reactions carried out by GPT in the cytoplasm (the glyoxylate-transamination reaction and the alanine-transamination reaction producing pyruvate and glutamate) increase their flux (Table S3).

The increase in the flux toward pyruvate and glutamate directly affects histidine metabolism because histidine can only be degraded to glutamate in the liver (or redirected to histamine but to a much lesser extent, because histamine is found in the nanomolar range whereas histidine is in the micromolar range). Specifically, according to the model, the increase in glutamate production results in increased flux through the urea cycle, allowing more histidine to be degraded to glutamate (Table S3; Supplemental Experimental Procedures). These results are consistent with the experimental results (Figure 1).

However, since the in silico metabolic model does not include reactions involved in intracellular histamine degradation (e.g., histamine N-methyltransferase [HMT] catalyzing the conversion of histamine into N-methyl-histamine), we cannot rule out an additional contribution of these reactions to the observed phenotype. Therefore, we measured N-methyl-histamine in *Agxt*^−/−^ mice, which was found to be increased compared to WT mice (Figure S2B).

To further probe the role of GPT in PH1, we also simulated GPT GoF in silico in the context of AGT LoF. As GPT carries out two reactions in the model (alanine transamination and glyoxylate transamination), we simulated an increase in the flux of both reactions. As reported in Table S4, the model predicts a reduction of oxalate levels induced by GPT GoF and a decrease in the histidine degradation flux. Unsurprisingly, if we simulate an increase only in the flux through the alanine-transamination reaction, but not through the glyoxylate-transamination reaction, then no reductions in oxalate levels and in histidine degradation are predicted to occur (Supplemental Experimental Procedures).

In conclusion, as shown in Figure 2, the reduction of histidine levels in PH1 is explained by an increase in the degradation of histidine in hepatocytes, mediated by GPT, and caused by an increased availability of alanine. The increased histidine degradation thus causes a decrease in histidine levels and hence in histamine levels.

### Alanine Reduces Histamine Levels in WT Mice while GPT Overexpression Restores Normal Histamine Levels and Reduces Urinary Oxalate in PH1 Mice

According to the in silico model, histidine/histamine reduction in AGT LoF occurs because alanine is redirected from AGT to GPT and thus secondarily alters histidine metabolism (Figure 2; Figure S1). If this is correct, then an excess of alanine in a WT context should cause a decrease of histamine levels systemically, whereas in PH1 mice there should be a weaker or no effect, as histamine is already reduced.

**Figure 3:**
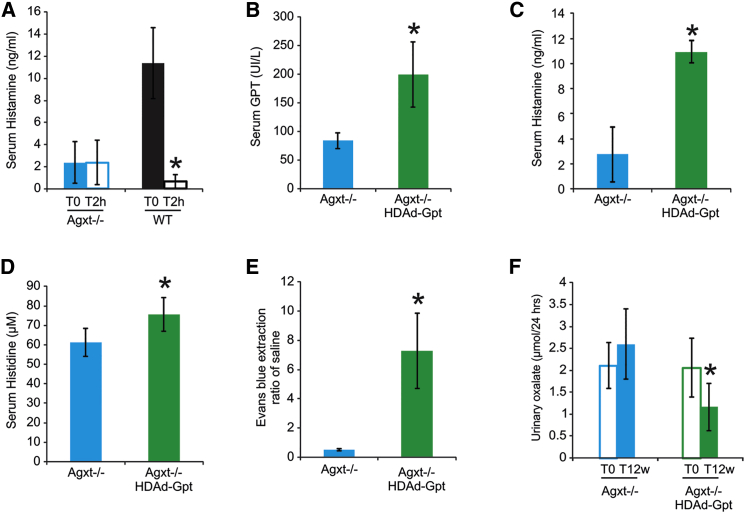
Alanine Administration Reduces Histamine Levels in WT Mice, while GPT Hepatic Overexpression Increases Histamine Levels and Vascular Permeability and Reduces Urinary Oxalate in *Agxt*^−/−^ Mice (A) Intraperitoneal injections of alanine reduced histamine levels at 2 hr after the injections (T2h) compared to baseline levels (T0) in WT mice (n = 5 per group). (B) Intravenous injection of HDAd-Gpt vector expressing the mouse *Gpt* gene under the control of a liver-specific expression cassette (Brunetti-Pierri and Ng, 2011, Brunetti-Pierri et al., 2005) resulted in high serum concentration of GPT protein in *Agxt*^−/−^ mice (n = 5 per group). (C and D) Serum histamine (C) and histidine (D) levels were significantly increased in GPT-overexpressing mice. (E) Vascular permeability measured as ratio of fluorescence units of Evans blue extracted from skin over saline after intradermal injection of 48/80 was increased in *Agxt*^−/−^ mice injected with HDAd-Gpt compared to controls (n = 8 per group). (F) Compared to baseline (T0) urinary oxalate was reduced at 12 weeks (T12w) post-injection in *Agxt*^−/−^ mice injected with HDAd-Gpt (E). ^∗^p < 0.05. See also Figures S1 and S2 and Table S4.

To validate this prediction, we investigated the effects of short-term alanine administration in mice. Intraperitoneal injection of alanine in WT mice resulted in decreased serum histamine levels at 2 hr compared to baseline levels, but no effects were detected in *Agxt*^−/−^ mice (Figure 3A; Figure S2C). The lack of response in *Agxt*^−/−^ mice was expected, since in these mice histamine levels are already low, while the flux through GPT is increased because of the AGT LoF. The experimental validation of this non-obvious prediction of the model supports the simplified model in Figure 2 linking glyoxylate detoxification to histidine metabolism.

To verify that GPT overexpression can restore normal histidine and histamine levels, as predicted in silico, at least when GPT can carry out also the *non-canonical* AGT-like reaction, we overexpressed GPT in *Agxt*^−/−^ mice by an HDAd-Gpt vector encoding the murine GPT enzyme under the control of a liver-specific expression cassette (Brunetti-Pierri and Ng, 2011, Brunetti-Pierri et al., 2005). *Gpt* gene transfer was confirmed by increased serum GPT levels (Figure 3B). Importantly, hepatic GPT overexpression increased serum histamine in PH1 mice (Figure 3C) to levels similar to those measured in WT mice (Figure 1B). Histidine, which was reduced in *Agxt*^−/−^ mice, increased following GPT overexpression (Figure 3D). Moreover, GPT overexpression increased vascular permeability in *Agxt*^−/−^ mice (Figure 3E). Finally, we also detected a decrease in urinary oxalate levels in HDAd-Gpt-injected *Agxt*^−/−^ mice compared to uninjected *Agxt*^−/−^ mice (Figure 3F), thus supporting GPT as a potential therapeutic target for PH1 therapy (Donini et al., 2009).

In conclusion, our in silico and experimental results support the simplified model of metabolism schematized in Figure 2 and Figure S1: the flux through the glutamate-pyruvate-transamination reaction carried out by GPT increases because alanine, which is no longer consumed by AGT, is redirected to GPT to produce pyruvate and glutamate. This perturbation in glutamate production has a direct effect on the flux through the histidine degradation pathway toward glutamate.

## Discussion

Mammalian intermediary metabolism was defined several years ago in biochemical terms from in vitro studies, but we are still lacking a clear picture of in vivo metabolism as a whole. Metabolic reactions are interconnected in a complex biochemical network where LoF of a single enzyme may result in perturbations of multiple, distant metabolic pathways. However, understanding how alterations in one pathway affect other pathways, which may appear remote from the initial metabolic defect, remains elusive.

Here, we show that human genome-scale metabolic models, coupled to appropriate computational approaches, can be effectively used to model in silico a liver IEM and to obtain clinically relevant results.

We demonstrated that deficiency of AGT unexpectedly results in reduced histidine and histamine levels, presumably through an increase in histidine catabolism mediated by GPT and caused by a redirection of the alanine flux from AGT in the peroxisome toward GPT in the cytosol. In agreement with the model predictions, we demonstrated in vivo in WT mice that increasing systemic levels of histidine results in increased serum levels of histamine, whereas increasing systemic levels of alanine results in decreased serum histamine.

A limited number of mammalian cells, namely mast cells and other immune cells, gastric enterochromaffin-like cells, and histaminergic neurons, express histidine decarboxylase, which is involved in histamine production. In the liver, hepatocytes, biliary epithelial cells, oval cells, and dendritic cells can convert histidine to histamine (Chang et al., 2010). Using a hepatocyte-derived cell line, we were indeed able to detect reduced histamine following AGT knockdown.

Our study suggests that molecules blocking AGT might reduce histamine systemically by depleting systemic levels of histidine, the substrate of histamine decarboxylase. Nevertheless, if developed, these antihistamine drugs should be used with caution because of potential oxalosis, which might occur because of severe and sustained reduction of AGT activity.

In addition, we demonstrated both in silico and in vivo that the increase in oxalate caused by AGT LoF is partly rescued by GPT overexpression. Hence, we confirmed GPT as a modifier gene or potential drug target for oxalate detoxification in patients with hyperoxalurias either primary or secondary. There have been great efforts to develop liver-directed gene therapy for IEMs (Piccolo and Brunetti-Pierri, 2015). Vectors encoding genes that are not functioning in disease state (i.e., *AGXT* in PH1) are obviously the first choice for gene therapy. Nevertheless, for PH1 patients harboring *null* mutations, who are at an increased risk for an immune reaction against the transgene product, gene therapy with the gene encoding for GPT would be an alternative option. Moreover, GPT expression can be combined within the gene therapy vector expressing AGT to further improve therapeutic efficacy.

Gene therapy with viral vectors has proven to be more difficult than initially anticipated, and immune system reactions against viral vectors and risks of insertional carcinogenesis remain important obstacles for clinical translation. Therefore, development of pharmacologic approaches based on small molecule drugs remains attractive. Toward this goal, the data generated in this study also could be exploited for therapy of PH1 by searching for human-approved drugs able to increase GPT expression. Besides applications for PH1 therapy, drugs reducing hyperoxaluria by GPT overexpression might be effective also for treatment of other forms of primary hyperoxalurias (e.g., PH2 and PH3) and more common forms of secondary hyperoxaluria (Xu et al., 2013). Our computational approach could be applied to identify additional drug-treatable targets to restore physiological levels of metabolites, which are altered in Mendelian or even complex multifactorial disorders. Indeed, in the case of PH1, it would be possible to identify enzymes whose overexpression or knockdown results in reduced oxalate levels.

Our work has implications for PH1 patients as well as for patients affected by other metabolic diseases. Genome-scale metabolic models painstakingly built up from scratch by the modeling research community (Gille et al., 2010, Jerby et al., 2010, Thiele et al., 2013) might be applied to a large number of disorders, potentially leading to new knowledge on disease pathogenesis and to the identification of therapeutic targets.

In summary, our results show that a single-enzyme LoF results in dysregulation of several metabolic reactions, going beyond the classic view on the pathogenesis of a Mendelian disease, which is narrowly focused on the affected pathway in which the enzyme operates.

More than a century ago, Archibald Garrod proposed the concept of “chemical individuality,” suggesting that even healthy individuals are biochemically unique due to inherited differences in enzymes, which are reflected in a predisposition to multifactorial diseases (Childs, 1970). In a more contemporary perspective, this concept is reflected in individualized medicine and metabolomic profiling that can detect individuals at higher risks of developing specific diseases to develop targeted preventive or therapeutic interventions. As metabolic profiling based on tandem mass spectrometry (MS/MS) and nuclear magnetic resonance (NMR)-based technologies is becoming more widely used for diagnostics, the major challenges facing metabolomics are to determine whether abnormal metabolites are indeed involved in pathogenesis or can act as disease biomarkers correlating with disease severity. Computational approaches based on genome-scale metabolic models might assist in interpreting and validating large-scale metabolic alterations.

## Experimental Procedures

### Extension and Analysis of the Genome-Scale Metabolic Model of Hepatocytes for PH1

The in silico metabolic network was described by a stoichiometric matrix (Pagliarini and di Bernardo, 2013) (Supplemental Experimental Procedures). A stoichiometric matrix contains information about all the metabolic transformations, and it is formed from the stoichiometric coefficients of each reaction that comprise the metabolic model, which commonly are integer numbers. Reactions and metabolites present in HepatoNet1 (Gille et al., 2010) network model were extended by including the following: (1) reactions and compounds known to be involved in glyoxylate metabolism, starting from published models (Duarte et al., 2007, Ma et al., 2007), public databases (Cerami et al., 2011, D’Eustachio, 2011, Kanehisa and Goto, 2000), and literature analysis (Cochat et al., 2012, Danpure, 1986, Donini et al., 2009); (2) transport reactions useful to balance the metabolic fluxes in the different compartments; and (3) metabolic routes, related to the amino acids that have been shown to be converted to oxalate, but which have not been completely defined yet (Asplin, 2002, Nguyen et al., 2001).

To validate the extended in silico model, we performed FBA to establish a flux distribution for each of the different metabolic objectives listed in Table S2. The principle of flux minimization (Holzhütter, 2004) was applied to optimize the metabolic objective and to compute an optimal metabolic flux, obtained as the solution of a constrained linear optimization problem. We also performed producibility analysis to check that the model was indeed able to produce all the compounds involved in glyoxylate metabolism. We defined a metabolite $x_{i}$ as producible if the network can sustain its synthesis under the steady-state and thermodynamic constraints. To test the producibility of $x_{i}$, we added a reaction in the cytoplasmic compartment consuming $x_{i}$, and then a flux-balance problem was solved to check if the in silico network was able to produce a strictly positive flux through this reaction. More details can be found in the Supplemental Experimental Procedures.

### Simulating Single-Enzyme LoF or GoF

To simulate the effect of an LoF of an enzyme $e_{j}$ catalyzing the reaction $r_{j}$, we solved 442 optimization problems, corresponding to the 442 metabolic objectives and physiological functions reported in Table S2, by constraining the fluxes through $r_{j}$ to zero. The same optimization problems were then solved for the WT model, but this time by constraining the fluxes through $r_{j}$ to be larger than an activity threshold. The results of these simulations were stored in two matrices that contained the metabolic fluxes of each reaction across the different metabolic objectives, in the WT and LoF simulations, respectively (Supplemental Experimental Procedures).

To simulate a GoF of $e_{j}$, we followed a similar procedure, but this time constraining the fluxes through $r_{j}$ to be greater at least ten times more than their value in the WT model (Supplemental Experimental Procedures).

### DFA and FVA

DFA is described in Pagliarini and di Bernardo (2013) and in the Supplemental Experimental Procedures. DFA aims at identifying the metabolites and reactions that are most affected by an enzyme LoF or GoF. Small molecules (e.g., water and carbon dioxide), cofactors (e.g., NADP and ATP), and a set of metabolites were not included, because they were involved in a large number of reactions (Table S2). DFA is based on computing FBA across 442 metabolic objectives for both the WT model and the perturbed model (i.e., with single-enzyme knockout or overexpression, or any combination of both). The average flux carried by each reaction across the 442 metabolic objectives for each of the two models is computed. For each reaction, we then take the difference of the average flux in the WT model minus its value in the modified model, expressed as a percentage of the WT flux. These differential fluxes are then used to rank the reactions from the ones that change the most in the modified model to the ones that change the least or do not change at all. Metabolites are then ranked according to the sum of the absolute values of the differential fluxes (Supplemental Experimental Procedures).

DFA allows us to compute the change in the concentration of a metabolite as result of an enzyme LoF or GoF, but not its sign. To establish if this concentration is expected to be elevated, reduced, or unchanged, we applied FVA (Duarte et al., 2007, Shlomi et al., 2009, Thiele et al., 2013). In FVA, an exchange interval for each metabolite is computed both in the WT model and in the LoF (GoF) model. This interval denotes the minimal flux and maximal flux of a metabolite that can be supported by the metabolic model. For each metabolite, FVA compares the exchange interval in WT model with the one in the LoF (GoF) model. If the two intervals overlap, then the metabolite is not affected by the enzyme LoF (GoF). Otherwise it is increased or decreased depending on the relative values of the two intervals (Supplemental Experimental Procedures). The code to run all of the simulations performed in this study can be found online (http://dibernardo.tigem.it).

### MSEA

MSEA is a bioinformatic tool to identify biologically meaningful patterns that are significantly enriched in metabolomic data and metabolic pathways (http://www.metaboanalyst.ca/) (Xia and Wishart, 2010). MSEA works by comparing the metabolites in a set to pre-defined functional groups. To identify the most likely diseases in Table S3, we used as input for the MSEA analysis the top 50 metabolites in Table S3 (Kyoto Encyclopedia of Genes and Genomes [KEGG] identifiers), and then we applied the over-representation analysis (ORA) of the MSEA online tool selecting the urine disease-associated metabolite sets option. To obtain the most significant pathways in Table S3, we used as input the top 20 metabolites in Table 1 and applied again the ORA, but this time selected the pathway-associated metabolite sets option. The list of pathways in Table S3 does not change significantly if we use the top 50 metabolites rather than the top 20.

### Mouse Procedures

*Agxt*^−/−^ mice were described previously (Salido et al., 2006) and animal procedures were performed in accordance to the regulation of the Italian Ministry of Health. *Agxt*^−/−^ mice were maintained on a SV-129 background. Injections of the HDAd vectors were performed in a volume of 200 μl in the retro-orbital plexus of 3-month-old male mice. Blood samples were collected by retro-orbital bleeding. SV-129 background age- and sex-matched mice were used as controls.

Histamine-mediated skin permeability was determined through a gross staining using the compound 48/80 (Enzo Life Sciences) as described previously (Donelan et al., 2006).

3-month-old male *Agxt*^−/−^ mice were injected intraperitoneally with L-Histidine (1 g/kg of body weight) and placed in metabolic cages for 24-hr urine collection. Blood samples for serum histamine levels were collected by retro-orbital bleeding 24 hr after histidine injections.

WT and *Agxt*^−/−^ 3-month-old male mice were injected intraperitoneally with L-Alanine (1 g/kg of body weight). Blood samples for serum histamine levels were collected by retro-orbital bleeding 2 hr after the alanine injections.

### HDAd Vectors

HDAd-AGT, HDAd-GPT, and HDAd-AFP vectors all bear the PEPCK-WL expression cassette (Brunetti-Pierri et al., 2005, Brunetti-Pierri et al., 2006), driving the expression of human *AGXT* or murine *Gpt*, respectively. HDAd was produced in 116 cells with the helper virus AdNG163 as described elsewhere (Pastore et al., 2013).

### Blood and Tissue Analyses

Histamine levels were determined in mouse serum and liver homogenates and in human plasma by ELISA (Labor Diagnostika Nord). For histamine determination on liver lysates, organs were homogenized in PBS using a Tissue Lyser (QIAGEN) and histamine levels were normalized for protein concentrations that were determined using Bradford Reagent (Bio-Rad).

Histamine in human plasma samples from PH1 patients and age-matched controls were analyzed by ELISA (IBL International). Methylhistamine was measured by ELISA (IBL International) on 24-hr urine collection according to the manufacturer’s protocol. Amino acids were measured by high-performance liquid chromatography (HPLC) at the Biochemical Laboratories at Baylor College of Medicine.

For western blotting, liver specimens were homogenized in radio-immunoprecipitation assay (RIPA) buffer and complete protease inhibitor cocktail (Sigma), incubated for 20 min at 4°C and centrifuged at 13,200 rpm for 10 min. Pellets were discarded and cell lysates were used for western blots. Proteins were loaded on a 12% SDS-PAGE, and, after transfer to a polyvinylidene fluoride (PVDF) membrane, blots were blocked with TBS-Tween-20 containing 5% non-fat milk for 1 hr at room temperature, followed by incubation with primary antibody overnight at 4°C. The primary antibodies used were a rabbit anti-human AGT (Sigma) and rabbit anti-calnexin (Enzo Life Sciences).

### Human Huh-7 Hepatic Cell Line

Huh-7 cells were cultured at 37°C with 5% CO_2_ in DMEM supplemented with 10% v/v fetal bovine serum and penicillin/streptomicin. Cells were transfected with a plasmid containing a small hairpin RNA (shRNA) for *AGXT* gene (OriGene) using TransIT-LT1 transfection reagent according to the manufacturer’s instructions (Mirus). Selection of transfected clones was performed starting at 24 hr after transfection with 1 μg/ml puromycin, and single stably transformed cell line clones were isolated. Downregulation of AGT was shown by western blotting with primary rabbit polyclonal anti-AGXT (Sigma) and primary monoclonal anti-calnexin antibody (Enzo Life Sciences). Horseradish peroxidase (HRP)-conjugated antibodies were used as secondary antibody (GE Healthcare).

### Statistical Analyses

Experimental data are presented as mean ± SD. Statistical significance was computed using the Student’s two-sided t test and p values < 0.05 were considered significant.

## Author Contributions

R.P., N.B.-P., and D.d.B. conceived the idea. R.P. designed the computational approach and performed simulations. F.N. helped with software coding, debugging, and testing. R.C. and N.B.-P. conceived and designed the experiments. R.C. performed the experiments. G.M. and M.D.M. provided human PH1 samples. R.B. generated the AGT-deficient cell line. P.A. generated the HDAd vectors. R.P., N.B.-P., and D.d.B. wrote the manuscript.

## Figures and Tables

**Table 1:** The Top 20 Metabolites of 590 Whose Concentrations Are Predicted to Be Altered the Most by DFA as a Result of AGT LoF

Rank (Differential Flux Balance)	Metabolite Name	KEGG ID	Predicted Change (FVA)
1	serine	C00065	−
2	alanine	C00041	=
3	pyruvate/hydroxypyruvate	C00022/C00168	−
*4*	*glyoxylate*	C00048	+
5	glycine	C00037	+
6	glycerate	C00258	−
*7*	*glycolate*	C00160	+
8	glycolaldehyde	C00266	=
9	glyceraldehyde	C02154	−
10	hypoxanthine	C00262	+
11	xanthine	C00385	=
12	glutamate	C00217	−
13	oxaloacetate	C00036	+
14	glutathione	C00051	=
15	glutathione disulfide	C00127	=
16	histidine	C00135	=
17	α-ketoglutarate	C00026	−
18	histamine	C00388	−
19	thymine	C00178	=
20	dihydrothymine	C00906	=

Metabolites are ranked by summing up the contribution of all differential fluxes involving each metabolite. The metabolites known to accumulate in the urine of PH1 patients (glyoxylate, glycolate, and oxalate) (Xia and Wishart, 2010) are shown in italic. Oxalate is ranked at position 39 of 590 (Table S3), hence it is not shown in this table. Column 4 reports the predicted sign of change in metabolite concentrations as computed by FVA (Supplemental Experimental Procedures): the metabolite concentration can be elevated (+), reduced (−), or (=) no sign could be predicted by FVA. Small molecules (e.g., water and carbon dioxide), co-factors (e.g., NADP and ATP), and a set of other metabolites were not considered because they are involved in a large number of reactions. The complete list of all metabolites and co-factors is reported in Table S2. Details on the analysis can be found in the Supplemental Experimental Procedures.

See also Figure S1 and Tables S2 and S3, which contains the complete list.

## Supplemental Information

Document S1. Supplemental Experimental Procedures, Figures S1–S3, and Table S1

Table S2. The 442 Metabolic Objectives Used for the In Silico Simulations Together with the Compounds, Small Metabolites, and Co-factor Excluded from the In Silico Analysis and the Demographics and AGXT Mutations of PH1 Patients, Related to Table 1 and Figure 2

Table S3. Metabolites and Reactions Whose Concentrations Are Affected by the AGT LoF According to In Silico Analysis and MSEA, Related to Table 1 and Figure 2

Table S4. Metabolites and Reactions Whose Concentrations Are Affected by Simulated GPT GoF In Silico in the Context of AGT LoF, Related to Figure 3 and the Experimental Procedures

Table S5. Metabolites and Reactions Whose Concentrations Are Affected by the AGT LoF According to In Silico and Forcing the Flux of Histidine to Histamine to Be Zero, Related to Figure 2 and the Experimental Procedures

Document S2. Article plus Supplemental Information
