# Supplementary material for: In Silico Modeling of Liver Metabolism in a Human Disease Reveals a Key Enzyme for Histidine and Histamine Homeostasis
Source: Cell Rep. 2016 May 26;15(10):2292–300. doi: 10.1016/j.celrep.2016.05.014 (PMC4906368; doi:10.1016/j.celrep.2016.05.014)
Supplement: Document S1. Supplemental Experimental Procedures, Figures S1–S3, and Table S1 [file mmc1.pdf]

Cell Reports, Volume *15*

Supplemental Information

In Silico Modeling of Liver Metabolism in a Human Disease Reveals a Key Enzyme for Histidine and Histamine Homeostasis

Roberto Pagliarini, Raffaele Castello, Francesco Napolitano, Roberta Borzone, Patrizia Annunziata, Giorgia Mandrile, Mario De Marchi, Nicola Brunetti-Pierri, and Diego di Bernardo

Supplemental Information: In silico modelling of liver metabolism in a human disease reveals a key enzyme for histidine and histamine homeostasis

Roberto Pagliarini[1,*], Raffaelel Castello[1,*],
Francesco Napolitano[1], Roberta Borzone[1], Patrizia Annunziata[1],
Giorgia Mandrile[2,3], Mario De Marchi[2,3]
Nicola Brunetti-Pierri[1,4,&] and Diego di Bernardo[1,5,&]

[1]Telethon Institute of Genetics and Medicine
80078 Pozzuoli (Naples), Italy
[2]Medical Genetics, San Luigi University Hospital
10043 Orbassano (Turin), Italy
[3]Department of Clinical & Biological Sciences
University of Turin, 10043 Orbassano (Turin), Italy
[4]Department of Translational Medicine
Federico II University, 80125 Naples, Italy
[5]Department of Chemical, Materials and Industrial Engineering
Federico II University, Naples, Italy

[*]equal contributors; [&]corresponding authors

1 Supplemental Figures

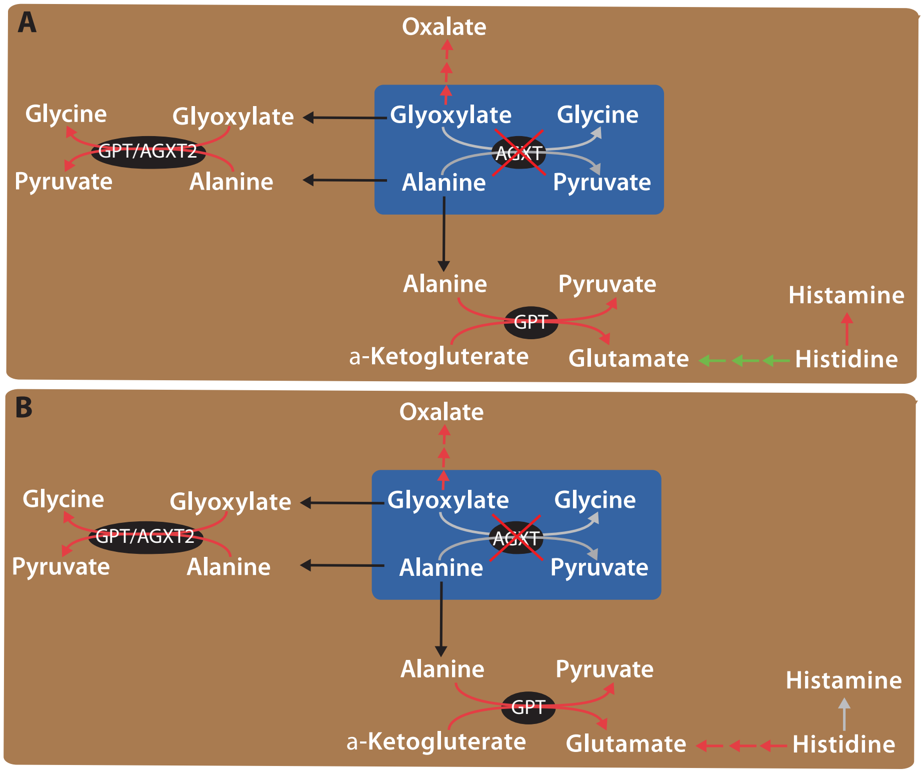

Figure S 1: **The link between AGXT Loss-of-Function and the histidine degradation pathway according to the *in silico* metabolic model. Related to Figures 1, 2, and 3 and Table 1**. Dashed lines represent pathways, while black ones represent transport reactions. Grey lines indicate Loss-of-Function (LoF), while green/red ones indicate a reduction/increase in the average metabolic flux across the 442 simulated metabolic objectives in each of the following conditions: (A) AGXT LoF versus Wild-Type: the metabolic fluxes through GPT and AGXT2 are increased, while the histidine degradation pathway towards glutamate is decreased. (B) AGXT LoF versus Wild-Type when forcing the flux of histidine to histamine reaction to be zero: the metabolic fluxes through GPT and AGXT2 are increased, but differently to (A), the histidine degradation pathway towards glutamate is increased.

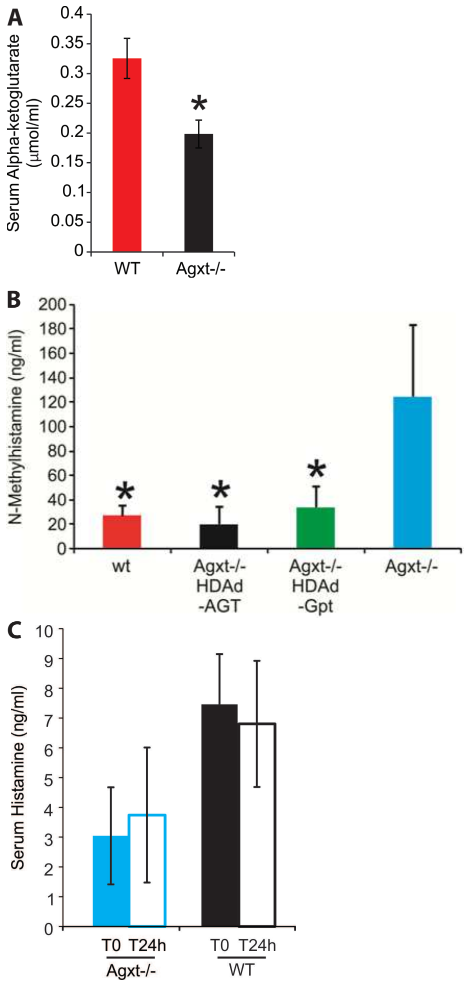

Figure S 2: **Metabolic alterations in PH1 mice. Related to Figures 1 and 3**. (A) $\alpha-$ketoglutarate reduction in Agxt$^{-/-}$ mice serum compared to control mice.$^{*}p < 0.05$. (B) N-methyl-histamine resulting from histamine degradation via histamine N-methyltransferase (HMT) was increased in Agxt$^{-/-}$ mice compared to wild-type (wt) mice and was normalized by the injections of either HDAd-AGT or HDAd-GPT. $^{*}p < 0.05$. (C) Short-term alanine administration has no effect on histamine levels 24hrs after administration. Intraperitoneal injections of alanine did not reduce histamine levels at 24 hours after the injections (T24h) compared to baseline levels (T0) in WT mice and PH1 mice (n=5 per group). On the contrary it strongly reduced histamine levels in WT mice at 2h after the injection as shown in **Figure 3A** of the main text. Indeed, alanine should be completely normalised in mice at 24hrs after the injection due to the high demand for alanine caused by the glucose-alanine cycle involving liver and muscles.

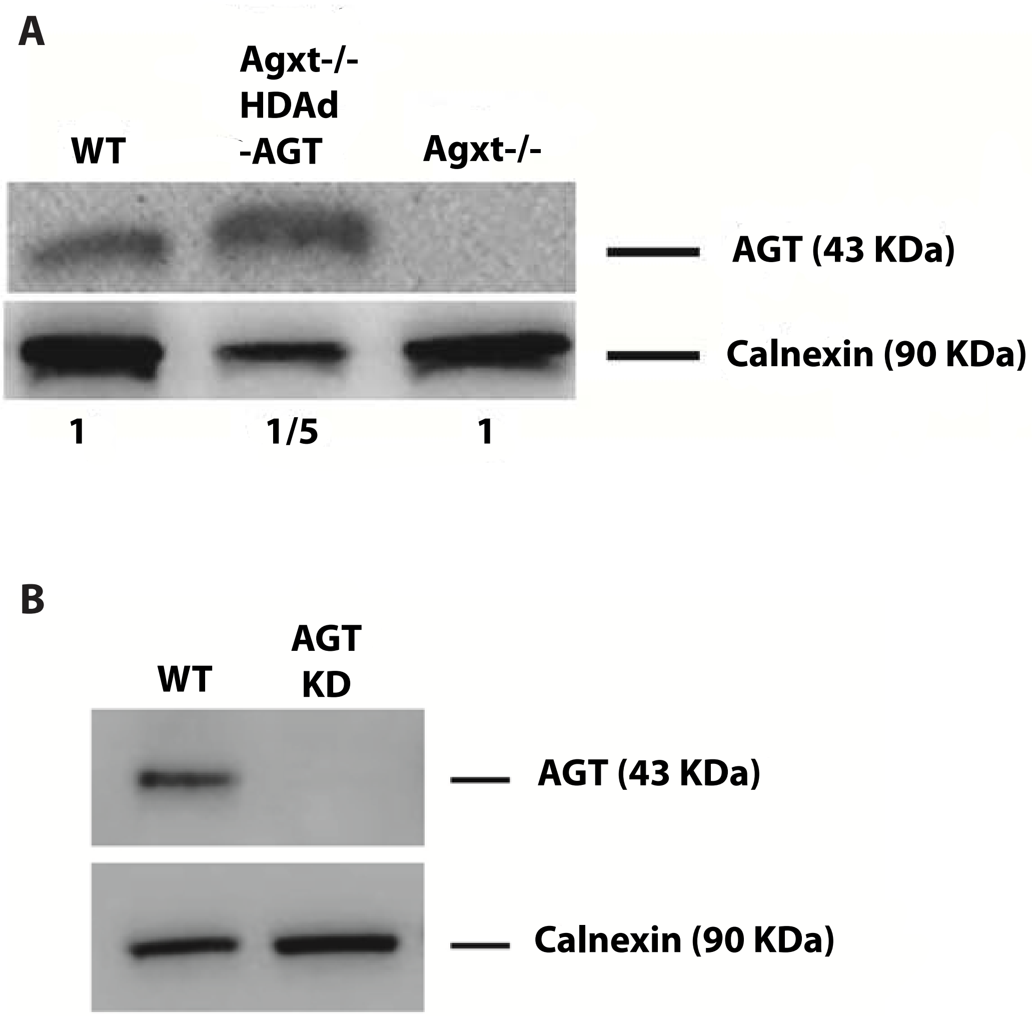

Figure S 3: **AGT protein levels in PH1 mice and Huh-7 cells. Related to Figure 1.** (A) Western blot of livers of Agxt$^{-/-}$ mice showed undetectable AGT protein that was increased after intravenous injection of HDAd-AGT vector expressing the human AGXT gene (protein amount loaded on gel was 1/5 in HDAd-AGT injected mice). (B) Western blot for AGT in AGT knock-down Huh-7 cells showed undetectable AGT protein levels.

2 Supplemental Tables

**Peroxisomal reactions**	**Ref.**
**Alanine(p)** + **Glyoxylate(p)** → **Glycine(p)** + Pyruvate(p)	[1,3,4]
**Serine(p)** + Pyruvate(p) → **Hydroxypyruvate(p)** + **Alanine(p)**	[3]
**Glyoxylate(p)** + O2(p) → H2O2(p) + **Oxalate(p)**	[1,3]
**Glycolate(p)** + O2(p) → **Glyoxylate(p)** + H2O2(p)	[3,6]
**Glycine(p)** + H2O(p) + O2(p) → **Glyoxylate(p)** + H2O2(p) + **NH3(p)**	[3,4]
Chenodeoxycholoyl-CoA(p) + **Glycine(p)** → CoA(p) + **Glycochenodeoxycholate(p)**	[8]
Choloyl-CoA(p) + **Glycine(p)** → CoA(p) + **Glycocholate(p)**	[8]
H2O(p) + O2(p) + **Sarcosine(p)** → Formaldehyde(p) + **Glycine(p)** + H2O2(p)	[8]
H2O2(p) ⇌ O2(p) + H2O(p)	[3]
**Serine(p)** + **Glyoxylate(p)** → **Hydroxypyruvate(p)** + **Glycine(p)**	[3]
**Cytoplasmic reactions**	**Ref.**
H2O2(c) ⇌ O2(c) + H2O(c)	[3]
**Glyoxylate(c)** + NAD+ (c) → NADH(c) + Oxalate(c)	[3,4]
**Glyoxylate(c)** + NADPH(c) → **Glycolate(c)** + NADP+(c)	[1,3]
**Glyoxylate(c)** + NADH(c) → **Glycolate(c)** + NAD+(c)	[3]
**3htmelys(c)** + H+(PG)(c) → **4tmeabut(c)** + Glycine(c)	[8]
**Gcald(c)** + H2O(c) + NAD+(c) → **Glycolate(c)**+ 2 H+(PG)(c) + NADH(c)	[8]
**Glyoxylate(c)** + Alanine(c) → Glycine(c) + Pyruvate(c)	[7]
Serine(c) ⇌ Glycine(c) + H2O(c)	[3]
**Glycolaldehyde(c)** + NAD+(c) → **Glycolate(c)** + NADH(c)	[3]
Hydroxypyruvate(c) → **Glycolaldehyde(c)** + CO2(c)	[3]
Hydroxypyruvate(c) + NADH(c) → Glycerate(c) + NAD+(c)	[1,3]
**Mitochondrial reactions**	**Ref.**
Alanine(m) + **Glyoxylate(m)** → Glycine(m) + Pyruvate(m)	[2,4]
**Glyoxylate(m)** + H+(PG)(m) + NADPH(m) → **Glycolate(m)** + NADP+(m)	[2]
Glycine(m) + H+(PG)(m) + Lipoamide(m) ⇌ **Alpam(m)** + CO2(m)	[8]
Glycine(m) + H+(PG)(m) + **Lpro(m)** ⇌ **ALpro(m)** + CO2(m)	[8]
FAD(m) + **Sarcosine(m)** + THF(m) → FADH2(m) + Glycine(m) + 5,10-Methylene-THF(m)	[8]
**Transport reactions**	**Ref.**
H2O2(c) ⇌ H2O2(p)	[*]
**NH3(p)** ⇌ NH3(c)	[*]
H+(PG)(p) ⇌ H+(PG)(c)	[*]
Alanine(c) ⇌ **Alanine(p)**	[*]
Pyruvate(p) ⇌ Pyruvate(c)	[*]
**Serine(p)** ⇌ Serine(c)	[*]
**Glycochenodeoxycholate(p)** ⇌ Glycochenodeoxycholate(c)	[*]
**Glycocholate(p)** ⇌ Glycocholate(c)	[*]
**Sarcosine(p)** ⇌ Sarcosine(c)	[*]
Formaldehyde(p) ⇌ Formaldehyde(c)	[*]
**NH4+(p)** ⇌ NH4+(c)	[*]
Glycine(c) ⇌ **Glycine(p)**	[2,6]
Glycine(c) ⇌ Glycine(m)	[2,6]
**Glycolate(c)** ⇌ **Glycolate(p)**	[1,4–6]
**Glycolate(c)** ⇌ **Glycolate(m)**	[*]
**Glyoxylate(c)** ⇌ **Glyoxylate(m)**	[4]
**Glyoxylate(c)** ⇌ **Glyoxylate(p)**	[1,4,5]
**Oxalate(p)** ⇌ Oxalate(c)	[5]
**Hydroxypyruvate(p)** ⇌ Hydroxypyruvate(c)	[12]
**Consuming reactions**	**Ref.**
Glycerate(c) →	[5]
Oxalate(c) →	[4]
**Glycolate(c)** →	[4]
**Glycolaldehyde(c)** →	[5]
Histamine(s) →	[*]

Table S 1: **The set of enzymatic, transport and consuming reactions that extends the genome-scale model developed in [9]. Related to Figure 2**. (c), (m), (b) represent metabolite compartment abbreviations: (c): cytosol; (m): mitochondrial matrix; (p): peroxisome. 3htmelys: 3-Hydroxy-N6,N6,N6-trimethyl-L-lysine, 4tmeabut: 4-Trimethylammoniobutanal, Alpam: S-aminomethyldihydrolipoamide, Alpro: S-Aminomethyldihydrolipoylprotein, Lpro: Lipoylprotein. The metabolite in bold font have been introduced in order to extend HepatoNet1. Ref. = references.

Table S 2 can be found in **Supplemental File 1**. **The 442 metabolic objectives used for the *in silico* simulations, together with the compounds, small metabolites and co-factor excluded from the *in silico* analysis and the demographics and AGXT mutations of PH1 patients. Related to Table 1 and Figure 2**. (Sheet1): the 442 metabolic objectives used for the in silico simulations. The metabolic objectives were adapted to our formulation of the linear optimisation problem (Subsection 3.1). Column 1: the unique name for the simulation, as in [9]; column 2: the target reactions; column 3: boundary reactions. MIMES, MIPES, PIPES, MES, DES, HES, WES define different sets of metabolites that can be taken up or released by the *in silico* network model. Further details about these sets can be found in [9]. (Sheet2): Compounds, small metabolites and co-factor excluded from the *in silico* analysis. (Sheet3): demographics and AGXT mutations of PH1 patients. *= this patient received kidney transplantation at 28 years.

Table S 3 can be found in **Supplemental File 2**. **Metabolites and reactions whose concentrations are affected by the AGT LoF according to *in silico* analysis and Metabolic Set Enrichment Analysis (MSEA). Related to Table 1 and Figure 2**. (Sheet1): reactions ranked according to DFA analysis. Reactions are ranked according to predicted mean absolute change of the flux in the 442 objective functions. Column 1: reactions; column 2: enzymes and transporters; column 3: the average flux value accross the 442 metabolic objective according to DFA; column 4: the absolute value of column 3 used to rank the reactions; columns 5 and 6 indicate the reactions direction in WT and PH1 conditions, respectively. In particular, if 0 the reaction is not active, if 1 the reaction direction goes from left to right, and if -1 the reaction direction goes from right to left. (Sheet2): compounds ranked considering model compartments. Metabolites are ranked according to the predicted absolute change, as computed by DFA using Eq. (7). (Sheet3): compounds ranked as in sheet 2 without considering and removing small molecules and cofactors listed in **Table S2**. (Sheet4): results from Metabolite Set Enrichment Analysis (diseases). Only diseases for which the MSEA p-value < 0.05 are reported. The top 50 metabolites predicted to change the most by the *in silico* model of PH1 (**Table S3**, Sheet3) have were used to run the analysis. Column Total indicates the number of metabolites in the set, while the column Hits reports the number of set compounds in the input list. (Sheet5): results from Metabolite Set Enrichment Analysis (pathways). Only pathways for which MSEA p-value < 0.05 are reported. The top 20 metabolites predicted to change the most by the *in silico* model of PH1 (**Table 1**) were used to run the analysis. Column Total indicates the number of metabolites in the set while, the column Hits reports the number of that compounds in the input list.

Table S 4 can be found in **Supplemental File 3**. **Metabolites and reactions whose concentrations are affected by simulated GPT GoF *in silico* in the context of AGT LoF. Related to Figure 3 and Experimental Procedure.** (Sheet1): reactions ranked according to DFA analysis. Reactions are ranked according to predicted mean absolute change of flux in the 442 objective functions. In particular. Column 1: reactions; column 2: enzymes and transporters associated with each reaction; column 3: the average value of flux across the 442 metabolic objective according to DFA; column 4: the absolute value of column 3 (the value used to rank the reactions); columns 5 and 6: reactions direction in WT and PH1 conditions, respectively. In particular, if 0 the reaction is not active, if 1 the reaction direction goes from left to right, and if -1 the reaction direction goes from right to left. (Sheet2): compounds ranked considering model compartments. Metabolites are ranked according to the predicted absolute change, as computed by DFA using Eq. (7). (Sheet3): compounds ranked as in sheet 2 without considering and removing small molecules and cofactors listed in **Table S2**.

Table S 5 can be found in **Supplemental File 4**. **Metabolites and reactions whose concentrations are affected by the AGT LoF according to *in silico* and forcing the flux of histidine to histamine to be zero. Related to Figure 2 and Experimental Procedure.** (Sheet1): reactions ranked according to DFA analysis. Reactions are ranked according to predicted mean absolute change of the flux in the 442 objective functions. Column 1: reactions; column 2: enzymes and transporters; column 3: the average flux value accross the 442 metabolic objective according to DFA; column 4: the absolute value of column 3 used to rank the reactions; columns 5 and 6 indicate the reactions direction in WT and PH1 conditions, respectively. In particular, if 0 the reaction is not active, if 1 the reaction direction goes from left to right, and if -1 the reaction direction goes from right to left. (Sheet2): compounds ranked considering model compartments. Metabolites are ranked according to the predicted absolute change, as computed by DFA using Eq. (7). (Sheet3): compounds ranked as in sheet 2 without considering and removing small molecules and cofactors listed in **Table S2**.

3 Supplemental Experimental Procedures

3.1 Constraint-based modelling of primary hyperoxaluria type I

The hepatocyte-specific metabolic network is described by a stoichiometric matrix $\mathbb{S} \in \mathbb{R}^{n\times 2m}$, where n is the number of metabolites and m the number of reactions. We considered $2m$ reactions because a metabolic flux can be, in principle, positive or negative depending on its direction (forward or reverse). To deal with non-negative variables, each reaction was thus represented by two irreversible ones (the forward and the backward reaction) [10].

The Flux Balance Analysis (FBA) problem can be stated as the solution for the vector variable V of the following equation:

$$\mathbb{S} \times V = 0 \tag{1}$$

where

$$V = (v_1^{(+)}, v_2^{(+)}, \ldots, v_m^{(+)}, v_1^{(-)}, v_2^{(-)}, \ldots, v_m^{(-)}) \in \mathbb{R}^{2m}$$

is the vector of fluxes associated with the forward and reverse reactions of the network.

Since in Eq. (1) usually $n < 2m$ the solution is not unique, hence a set of physiological meaningful constraints is added to the possible values that the metabolic fluxes V can achieve. Moreover, it is necessary to define input and output metabolites, i.e. the set of boundary reactions ($\mathcal{R}_{bound}$) specifying which metabolites are provided to the network (inputs) and which ones have to be produced (outputs). These boundary reactions are needed to obtain physiological meaningful solutions. For a physiological function with k boundary reactions, we can rewrite Eq. (1) as an extended stoichiometric matrix $\tilde{\mathbb{S}} \in \mathbb{R}^{n\times(2m+k)}$ and an extended vector of fluxes $\tilde{V} \in \mathbb{R}^{2m+k}$. Finally, in order to simulate a specific physiological function (i.e. breakdown of an amino acid to produce glucose) a set of target reactions ($\mathcal{R}_{tar}$) with a fixed flux value has to be specified.

The FBA problem can thus be formally stated as the solution to the following linear optimisation problem [10]:

$$\min_{V\in\mathbb{R}^{2m}} \left(\sum_{j=1}^{m} (w_j \times v_j^{(+)} + w_j \times K_j^{equ} \times v_j^{(-)}) \right) \tag{2}$$

subject to the following constraints:

$$\begin{array}{rcl} \tilde{\mathbb{S}} & \times & \tilde{V} = 0 \\ l_j^{(+)} \leq v_j^{(+)} \leq u_j^{(+)} & if & r_j \notin \mathcal{R}_{tar} \\ l_j^{(-)} \leq v_j^{(-)} \leq u_j^{(-)} & if & r_j \notin \mathcal{R}_{tar} \\ v_j = k_j & if & r_j \in \mathcal{R}_{tar} \end{array} \tag{3}$$

where $u_j^{(+)}, u_j^{(-)} \in \mathbb{R}^+$ represent the upper bounds of $v_j^{(+)}$ and $v_j^{(-)}$, respectively, and $l_j^{(+)} = l_j^{(-)}$ the lower bounds (we set $l_j^{(+)} = l_j^{(-)} = 0$ in our simulations); w_j is the weight associated with the flux v_j; the equilibrium constants K_j^{equ} have been

introduced to constraint fluxes according to Gibbs free energy calculations as listed in supplementary data of [9]. Weighting the backward flux with the thermodynamic equilibrium constants takes into account the thermodynamic effort connected with reversing the natural direction of the reaction [10].

Specifically, we expressed the equilibrium constant for a reaction r_j through the change of Gibbs free energy under the standard conditions, denoted as $\Delta G^0_{r_j}$, by applying the following rule:

$$K_j^{equ} = e^{-\frac{\Delta G^0_{r_j}}{R \cdot T}} \tag{4}$$

where R is the universal gas constant and T is the absolute temperature, that we set to $37°C$ in order to model the normothermia. All the values for the constants in Eq. (2) and (3) were chosen as in [9].

In order to validate the extended HepatoNet1 metabolic network model (i.e. including glyoxylate related reactions), we first performed flux-balance analyses, as described above, to establish a flux distribution for each of the different metabolic objectives listed in **Table S2**. We then applied a *producibility analysis* to test that the model was indeed able to produce all the compounds in glyoxylate metabolism. We define a metabolite x_i as *producible* if the network can sustain its synthesis under the steady state and thermodynamic constraints. To test the producibility of x_i, we added a reaction r_j in the cytoplasmic compartment which consumes x_i, and then solved the flux-balance problem to check if the network was able to produce a strictly positive flux through r_j.

3.2 Simulation of the loss- or gain-of-function of an enzyme with the metabolic network model

In order to simulate the effect of a loss-of-function (LoF) of an enzyme e_j catalyzing the reaction r_j, we first solved $l = 442$ optimization problems of type (2) to compute the wild-type flux distributions across 442 different metabolic conditions (a list of the metabolic objectives is reported in **Table S2**) forcing the fluxes through enzyme e_j to be greater than a threshold. Secondly, we computed the LoF flux distributions by solving the same 442 flux-balance problems but this time constraining the fluxes through enzyme e_j to zero, that is, $v_j^{(+)} = v_j^{(-)} = 0$. The results of the simulations were stored in two matrices that contain the values of the fluxes for each of the m internal reactions computed either in the wild-type simulations ($\mathbb{V}^{wt} \in \mathbb{R}^{m \times l}$,) or in the loss of function simulations ($\mathbb{V}^{ko} \in \mathbb{R}^{m \times l}$). Namely, $v_{i,j}^{wt} \in \mathbb{V}^{wt}$ ($v_{i,j}^{ko} \in \mathbb{V}^{ko}$) represents the flux of reaction r_i in the j-th metabolic functions, with $v_{i,j} = v_i^{(+)}$ if $v_i^{(+)} > 0$ and $v_{i,j} = -v_i^{(-)}$ if $v_i^{(-)} > 0$. We followed this rule to store the value of a metabolic flux v_i and to take the direction of r_i into account.

In order to simulate the effect of a gain-of-function (GoF) of an enzyme e_j catalyzing the reaction r_j we adopted the following rule: if $\Delta G^0_{r_j} \leq 0$ then $u_j^{GoF(+)} = u_j^{(+)} + k$ and $l_j^{GoF(+)} = k$, otherwise $u_j^{GoF(-)} = u_j^{(-)} + k$, $l_j^{GoF(-)} = k$, with $k = 1000$. Effectively these constrains force the flux through enzyme e_j in the GoF simulation to be increased of k compared to the wild-type model. As for the LoF, the

results of the simulations were stored in a matrix $\mathbb{V}^{GoF}$.

3.3 Differential Flux-balance Analysis (DFA)

We first applied a computational approach we previously described in [13]. Specifically, for each reaction r_i we computed the difference ($\delta_{i,j}$) between the flux in the wild-type model and the flux in the LoF (or GoF) model for the $j-th$ metabolic objectives (**Table S2**):

$$\Delta = \mathbb{V}^{wt} - \mathbb{V}^{ko} \in \mathbb{R}^{m \times l} \tag{5}$$

where $\delta_{i,j}$ is the element of Δ having indexes i and j. Next, we computed for each reaction r_i the average flux difference across the l metabolic objectives :

$$\delta_i = \frac{1}{l}\sum_{j=1}^{l} \delta_{i,j} \tag{6}$$

for $i = 1, 2, \ldots m$. These values are used to rank each reaction, arranged in descending order. In this way, reactions at the top of the list are those predicted to have a reduced metabolic flux because of the enzyme loss-of-function (or gain-of-function), vice-versa those at the bottom of the list, will be the reactions whose flux is predicted to increase as consequence of the enzyme loss-of-function (or gain-of-function).

In order to rank metabolites, we also took into account the stoichiometry of the metabolic network to estimate the impact of the enzyme LoF (or GoF) on a metabolite concentration x_i by means of the following index:

$$\psi_{x_i} = \sum_{j=1}^{m} \hat{s}_{i,j} |\delta_i| \tag{7}$$

where $\hat{s}_{i,j}$ is the element of matrix $\hat{\mathbb{S}}$, the binary form of $\tilde{\mathbb{S}}$ [14], of index (i, j). The $\psi_{x_1}, \psi_{x_2}, \ldots, \psi_{x_n}$ values are used to obtain a ranked list of metabolites (X^{ord}, **Table 1 and Table S3**). This list reports, at the top, the metabolites most affected by a loss-of-function (or gain-of-function). Small molecules (e.g., water, carbon dioxide), cofactors (e.g., NADP, ATP), and a set of compounds were removed, because they are involved in a large number of reactions (**Table S2**).

3.4 Flux Variability Analysis (FVA)

DFA estimates how much the fluxes involving a specific metabolite change in response to a LoF (or GoF) of a specific enzyme. We thus take this quantity (i.e. the value computed as in Eq. (eq:metrank)) as a proxy for the potential change in metabolite concentration as result of the enzyme LoF (or GoF). However DFA canno predict the sign of the change (i.e. whether the metabolite is elevated or reduced). This happens because FBA assumes that no metabolites' accumulation or depletion can occur.

In order to overcome this limitation, we applied Flux Variability Analysis (FVA) as described in [11, 15, 16]. The idea is to compute an exchange interval I for each

metabolite x both in the WT model and in the LoF (or GoF) model. This interval denotes the minimal (v_{min}) and maximal (v_{max}) output flux of a metabolite x which can be supported by the metabolic model. For each metabolite, we compared the exchange interval computed for the WT model to the one computed for the LoF (GoF) model. If the two intervals coincide, then no sign can be assigned to the metabolite. Otherwise it is said to increase or decrease depending on the values of the two intervals.

In order to compute the exchange interval $I_{cond} = [v_{min}, v_{max}]$ for a metabolite x in condition $cond \in \{WT, LOF\}$ we proceeded as follows: we first introduced, if not yet in the model, a consuming reaction r in the cytoplasm associated to a flux v consuming the metabolite x; an exchange interval $I^k_{cond} = [v^k_{min}, v^k_{max}]$ for x in each of the $k = 1 \dots 442$ metabolic objectives is then computed by determining the minimal and maximal values of v^k by solving the following two linear programming optimization problems, subject to the constrains in Eqs. (3), for the appropirate model (e.g. WT or LOF):

$$\min_{V \in \mathbb{R}^{2m}} \left(\frac{1}{2} \left(\sum_{j=1}^{m} (w_j \times v_j^{(+)} + w_j \times K_j^{equ} \times v_j^{(-)}) \right) + \frac{1}{2} v^k_{min} \right) \tag{8}$$

in order to obtain v^k_{min} and

$$\min_{V \in \mathbb{R}^{2m}} \left(\frac{1}{2} \left(\sum_{j=1}^{m} (w_j \times v_j^{(+)} + w_j \times K_j^{equ} \times v_j^{(-)}) \right) - \frac{1}{2} v^k_{max} \right) \tag{9}$$

in order to obtain v^k_{max}.

The exchange interval of the metabolite x is then defined as

$$I_{cond} = \left[\max_{k \in [1,442]} (v^k_{min}), \max_{k \in [1,442]} (v^k_{max}) \right].$$

The results of DFA and FVA to the PH1 model are reported in the main manuscript (**Table 1**) and in the **Supplemental information** (**Table S3**).

3.5 *In silico analysis* of AGT Loss of Function

We applied DFA to the extended Hepatonet1 model in order to rank all of the metabolites ($\approx$ 800) and reactions in the model ($\approx$ 2600) according to their average magnitude of change in the AGT LoF versus wild-type model across the 442 physiological objectives. The reactions are reported in **Table S3**, ranked according to the absolute change, and metabolites are reported in **Table S3** and **Table 1** of the main manuscript.

As summarised in **Figure 2**, **Figure S1A**, and **Table S3**, the GPT reaction increases its flux because of an excess of alanine, which is no longer consumed by the AGT reaction in the peroxisome and it is thus redirected to GPT in the cytoplasm. The increase in flux through GPT, which produces pyruvate and glutamate, is thus counterbalanced by a **decrease** in the histidine degradation flux towards glutamate (**Figure S1A**). To

achieve this reduction, the model forces the flux from histidine to histamine to increase (**Figure S1A**). This scenario however is not physiological for several reasons: (1) the physiological concentrations of histamine (nanomolar range) and histidine (micromolar range) are very different; (2) amino-acid degradation in the liver mainly occurs to produce energy in the form of glucose and not to produce histamine.

Therefore, we performed additional simulations for each of the 442 metabolic objectives listed in **Table S2**, this time forcing the flux of histidine to histamine to be zero (**Table S5**). As before, the PH1 model predicts an increased flux through GPT caused by an excess of alanine because of AGT loss of function. However, this time the histidine degradation flux to glutamate cannot be reduced (since histidine has nowhere else to go). Therefore, as depicted in (**Figure S1B**) and reported in **Table S5**, the model finds another solution to balance glutamate excess, i.e. it increases the flux from histidine to glutamate, and at the same time, increases glutamate metabolism and the urea cycle, so that glutamate levels are kept in check. In this case, the reduction of histidine/histamine levels is explained by the *in silico* model by an increased degradation of histidine in hepatocytes.

We believe the latter solution to be the most physiological plausible, and in agreement with our experimental observation of reduced histidine levels systemically in PH1 mice and patients, and of reduced histamine levels in wild-type mice following acute alanine administration.

Regardless of which solution is the most physiologically relevant, both solutions suggests a central role of alanine and the GPT enzyme in mediating the effects of the AGT LoF on histidine metabolism.

References

[1] J. R. Asplin. Hyperoxaluric calcium nephrolithiasis. *Endocrinol Metab Clin North Am*, 31(4):927–49, 2002.

[2] P. R. S. Baker, S. D. Cramer, M. Kennedy, D. G. Assimos, and R. P. Holmes. Glycolate and glyoxylate metabolism in hepg2 cells. *American Journal of Physiology - Cell Physiology*, 287(5):C1359–C1365, 2004.

[3] B. Vogelstein Editor K. W. Kinzle Editor S. E. Antonarakis Editor A. Ballabio Editor K. M Gibson Editor G. Mitchel Editor D. Valle Editor-in Chief, A. L. Beaudet Editor. *The Online Metabolic and Molecular Bases of Inherited Disease, Chapter 133*. Mc Graw Hill, International Edition.

[4] C. J. Danpure. Primary hyperoxaluria type 1: Agt mistargeting highlights the fundamental differences between the peroxisomal and mitochondrial protein import pathways. *Biochimica et Biophysica Acta (BBA) - Molecular Cell Research*, 1763(12):1776 – 1784, 2006.

[5] C. J. Danpure and G. Rumsby. Molecular aetiology of primary hyperoxaluria and its implications for clinical management. *Expert Reviews in Molecular Medicine*, 6:1–16, 2004.

[6] C.J. Danpure and P.R Jennings. Peroxisomal alanine:glyoxylate aminotransferase deficiency in primary hyperoxaluria type i. *FEBS Letters*, 201(1):20 – 34, 1986.

[7] S. Donini, M. Ferrari, C. Fedeli, M. Faini, I. Lamberto, A. Serena Marletta, L. Mellini, M. Panini, R. Percudani, L. Pollegioni, L. Caldinelli, S. Petrucco, and A. Peracchi. Recombinant production of eight human cytosolic aminotransferases and assessment of their potential involvement in glyoxylate metabolism. *Biochemical Journal*, 422(2):265–272, 2009.

[8] N. C. Duarte, S. A. Becker, N. Jamshidi, I. Thiele, M. L. Mo, T. D. Vo, R. Srivas, and B. Palsson. Global reconstruction of the human metabolic network based on genomic and bibliomic data. *Proceedings of the National Academy of Sciences*, 104(6):1777–1782, February 2007.

[9] C. Gille, C. Bolling, A. Hoppe, S. Bulik, S. Hoffmann, K. Hubner, A. Karlstadt, R. Ganeshan, M. Konig, K. Rother, M. Weidlich, J. Behre, and H. Holzhutter. HepatoNet1: a comprehensive metabolic reconstruction of the human hepatocyte for the analysis of liver physiology. *Molecular Systems Biology*, 6(1), September 2010.

[10] H. G. Holzhütter. The principle of flux minimization and its application to estimate stationary fluxes in metabolic networks. *European Journal of Biochemistry*, 271(14):2905–2922, 2004.

[11] L. Jerby, T. Shlomi, and E. Ruppin. Computational reconstruction of tissue-specific metabolic models: application to human liver metabolism. *Molecular Systems Biology*, 6(1), 2010.

[12] C. G. Monico, M. Persson, G. C. Ford, G. Rumsby, and D. S. Milliner. Potential mechanisms of marked hyperoxaluria not due to primary hyperoxaluria I or II. *Kidney International*, 62:392–400, 2002.

[13] R. Pagliarini and D. di Bernardo. A genome-scale modeling approach to study inborn errors of liver metabolism: Toward an in silico patient. *Journal of Computational Biology*, 20(5):383–397, 2013.

[14] B. O. Palsson. *Systems Biology: Properties of Reconstructed Networks*. Cambridge University Press, 1 edition, January 2006.

[15] T. Shlomi, M. N. Cabili, and E. Ruppin. Predicting metabolic biomarkers of human inborn errors of metabolism. *Molecular Systems Biology*, 5, April 2009.

[16] I. Thiele, N. Swainston, R. M. T. Fleming, A. Hoppe, S. Sahoo, M. K. Aurich, H. Haraldsdottir, M. L. Mo, O.r Rolfsson, M. D. Stobbe, S. G. Thorleifsson, R. Agren, C. Bolling, S. Bordel, A. K. Chavali, P. Dobson, W. B. Dunn, L. Endler, D. Hala, M. Hucka, D. Hull, D. Jameson, N. Jamshidi, J. J. Jonsson, N. Juty, S. Keating, I. Nookaew, N. Le Novere, N. Malys, A. Mazein, J. A. Papin, N. D. Price, E. Selkov, M. I. Sigurdsson, E. Simeonidis, N. Sonnenschein, K. Smallbone, A. Sorokin, J. H. G. M. van Beek, D. Weichart, I. Goryanin, J. Nielsen, H. V. Westerhoff, D. B. Kell, P. Mendes, and B. O. Palsson. A community-driven global reconstruction of human metabolism. *Nature Biotechnology*, 31(5):419–425, March 2013.
